# Comparison of Transforaminal Percutaneous Endoscopic Lumbar Discectomy with and without Foraminoplasty for Lumbar Disc Herniation: A 2-Year Follow-Up

**DOI:** 10.1155/2019/6924941

**Published:** 2019-01-02

**Authors:** Binbin Wu, Gonghao Zhan, Xinyi Tian, Linyu Fan, Chenchen Jiang, Beekoo Deepti, Hong Cao, Jun Li, Qingquan Lian, Xixi Huang, Feng Xu

**Affiliations:** ^1^Department of Pain Medicine, The Second Affiliated Hospital and Yuying Children's Hospital of Wenzhou Medical University, Wenzhou, Zhejiang, China; ^2^China-USA Neuroimaging Research Institute, The Second Affiliated Hospital and Yuying Children's Hospital of Wenzhou Medical University, Wenzhou, Zhejiang, China; ^3^Department of Clinical Research Center, The Second Affiliated Hospital and Yuying Children's Hospital of Wenzhou Medical University, Wenzhou, Zhejiang, China; ^4^Department of Anesthesiology, The Second Affiliated Hospital and Yuying Children's Hospital of Wenzhou Medical University, Wenzhou, Zhejiang, China

## Abstract

**Background:**

Both transforaminal percutaneous endoscopic lumbar discectomy with foraminoplasty (TF PELF) and transforaminal percutaneous endoscopic lumbar discectomy without foraminoplasty (TF PELD) were developed for lumbar disc herniation (LDH) patients. However, the safety and effectiveness between the TF PELF and TF PELD have not been investigated.

**Methods:**

Of the included 140 LDH patients, 62 patients received TF PELF (PELF group) and 78 patients received TF PELD (PELD group). The operation time, the duration of staying at the hospital, and complication incidences were recorded. All patients were followed up for 2 years, where low back and leg visual analogue scale (VAS) pain ratings and Oswestry Disability Index (ODI) were compared between the 2 groups before and after surgery. Modified Macnab criterion was estimated for all patients at postoperative 2 years.

**Results:**

There were no significant difference of the operation time, number of days staying at the hospital, and the incidence of complications between the 2 groups (*P* > 0.05). Two cases in the PELF group and 1 case in the PELD group received a second surgery due to unrelieved symptoms postoperatively. Low back and leg VAS and ODI scores decreased in both groups after operation (*P* < 0.01), respectively, but were not significant between the 2 groups over time (*P* > 0.05). Six patients in the PELF group and 3 patients in the PELD group did not continue the follow-up; thus, only 131 patients completed Macnab evaluation. The satisfactory rate was reported as 80.4% in the PELF group and 90.7% in the PELD group (*P* > 0.05).

**Conclusions:**

This study suggested that the safety and effectiveness of TF PELF are comparable to TF PELD for LDH patients.

## 1. Introduction

Low back pain was reported affecting up to 80% of the population during their lifetime [1], disabling 5–10% of the people, which is a major concern and accounts for up to 75–90% of the cost [2], and was the top source of disability and lost productivity in the United States [3]. Lumbar disc herniation (LDH) is a widespread medical problem, closely associated with low back and leg pain mostly affecting 30- to 50-year-old people. It was also reported that 80% adults suffered from low back and/or leg pain at least once in their life time, and of these patients in China, around 20% were caused by LDH [4].

Open lumbar discectomy was implemented as a standard surgery for LDH therapy, firstly described by Dandy and Peltier [5] The improvement of minimally invasive methods was achieved after introduced with the microscope, and microscope discectomy has been the predominant surgical approach for LDH during past decades.. However, minimally invasive surgery is gaining increasing attention, including in the area of spinal surgery. The anatomic neural foramen described by Kambin with the purpose for endoscopic access was seemed as a cornerstone in the development of a fully endoscopic transforaminal approach, which was followed by the endoscopic spine system introduced by Yeung [6]. After that, three different operative approaches of percutaneous endoscopic lumbar discectomy (PELD) were mainly developed gradually, including interlaminar, TF, and posterolateral discectomy. The TF approach is the most popular, used with the advantage of ensuring safety in “Kambin's” triangle [7].

The overall success rate of conventional microdiscectomy ranged from 75 to 100% [8] and that of transforaminal PELD was 69–90% [9–12]. Therefore, transforaminal PELD might be an important alternative to conventional open microdiscectomy, and their clinical outcomes were reported to be comparable [13–15]. Moreover, transforaminal PELD can be operated under local anesthesia. Hence, this procedure is possible for elderly patients with poor general conditions and provides better feedback to avoid potential nerve root damage from manipulation during operation [16] with advantages of small incision size, limited blood loss, less surrounding tissue injury, rapid recovery, short hospital stay, and less postoperative pain [14].

Recently, the significance of foraminoplasty has been widely emphasized. It was defined as “widening the foramen by undercutting the ventral part of the superior articular process (SAP) with ablation of foraminal ligament with the use of bone trephines, endoscopic drill, and side-firing laser to visualize the anterior epidural space and its contents” [17]. The working place can be enlarged, and the cannula can be navigated through a very narrow space, allowing the removal of the herniated mass completely without injuring the exiting nerve root [18]. Transforaminal PELD with foraminoplasty (TF PELF) was reported safe for the patients and reached a satisfactory rate of 92.5% [12]. Whereas, in addition to the disadvantages of bleeding, pain, and extended operation time, foraminoplasty also would cause postoperative flares with an incidence of 19% [19]. Furthermore, 6.1% patients complained of dysesthesia [20], which might be attributed to increasing temperature when using a side-firing laser or high-speed drill, hence, leading to nerve inflammation and deterioration of nerve conduction to some extent [21].

Upon the development of PELD, a technique of transforaminal PELD without foraminoplasty (TF PELD) was adopted on treating LDH. However, whether the injury during the TF PELF procedure would deteriorate the clinical outcomes compared with TF PELD on LDH treatment is an open question. In the present study, 140 patients with LDH who underwent TF PELF (62 cases, PELF group) or TF PELD (78 cases, PELD group) were recruited. The authors comprehensively compared the postoperative clinical outcomes between the two groups with a 2-year follow-up.

## 2. Materials and Methods

### 2.1. Patients

With approval from the Institutional Review Board of the Second Affiliated Hospital and Yuying Children's Hospital of Wenzhou Medical University, the study population comprised 140 consecutive patients with LDH who underwent TF PELF or TF PELD surgery in our department from July 2014 to August 2016. All of the patient met the inclusion criteria and were followed up to 2 years postoperatively. All the patients provided the informed consents and protocols that described the details of the follow-up.

**Figure 1:**
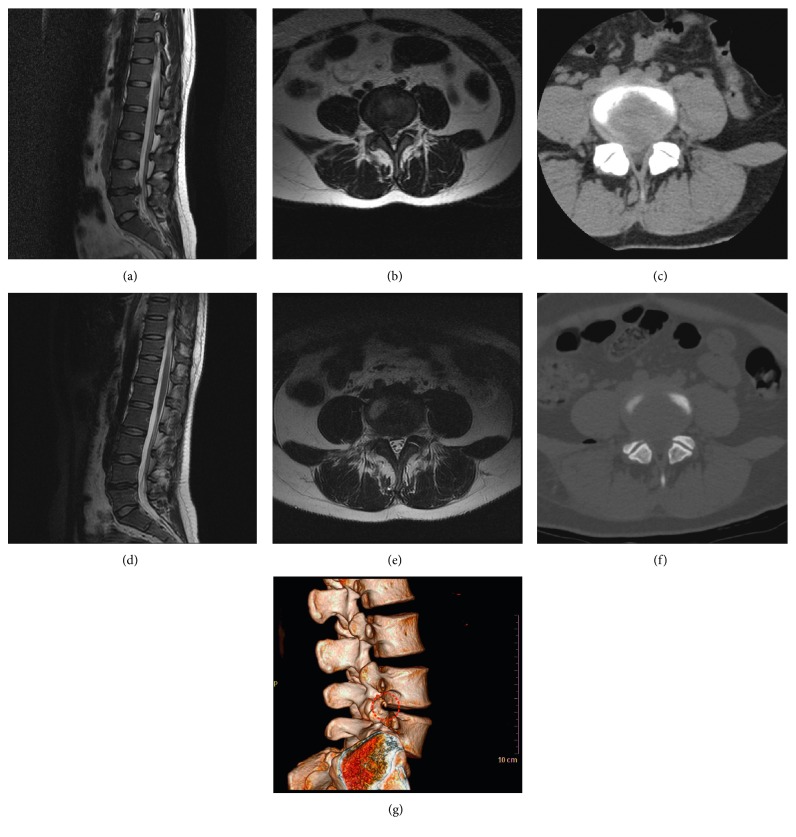
The preoperative and postoperative imaging data of patient who received TF PELF. (a and b) A preoperative MRI image shows the sagittal and coronal views of a patient diagnosed with LDH. (c) A preoperative CT image of the same patient. (d and e) The postoperative MRI sagittal and coronal views of the patient after TF PELF. (f) A postoperative CT image. (g) The postoperative 3D CT imaging result. The red round denotes foraminoplasty.

**Figure 2:**
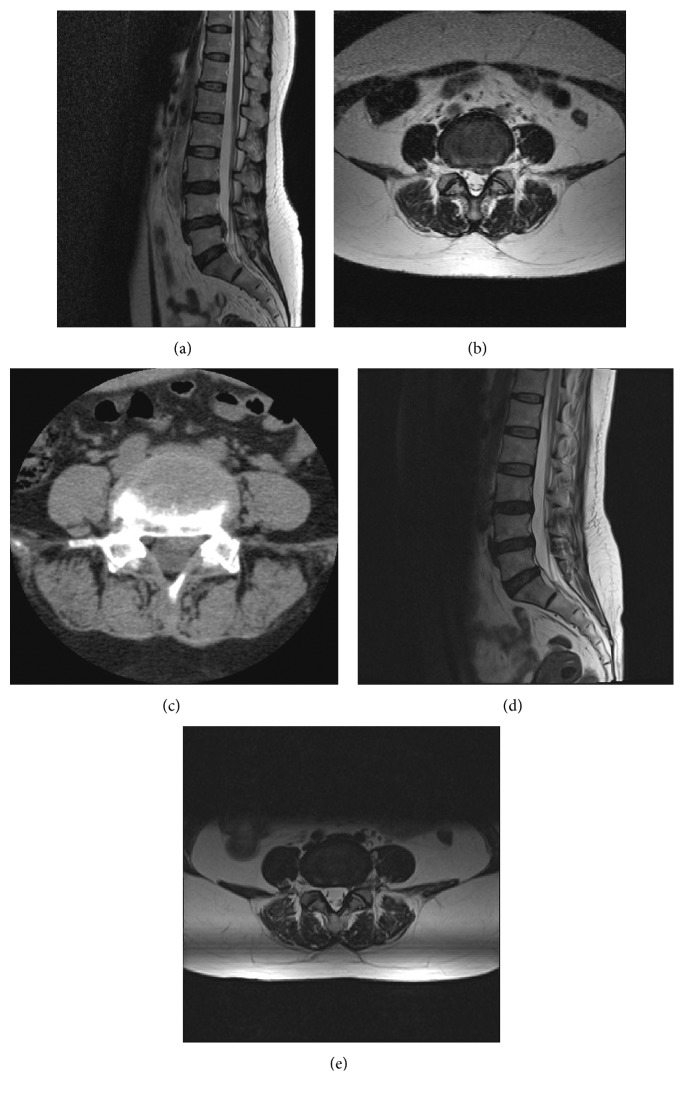
The preoperative and postoperative imaging data of patient who received TF PELD. (a and b) A preoperative MRI image shows the sagittal and coronal views of a patient diagnosed with LDH. (c) A preoperative CT image of the same patient. (d and e) The postoperative MRI sagittal and coronal views of the patient after TF PELD.

Inclusion criteria were as follows: (1) preoperative imaging evidence of LDH at L1-2, L2-3, L3-4, L4-5, and L5-S1 (monosegmental or double segmental), with or without canal and/or lateral recess stenosis caused by herniated mass on magnetic resonance images (MRI) and computed tomography (CT) (Figures 1 and 2). (2) Presented with symptoms of lumbar radiculopathy with low back pain, leg pain, decreased motor function, and/or dysesthesia, which was in accordance with the presentation of MRI and CT. (3) Dynamic flexion-extension radiographs, the neutral anterior-posterior, and lateral radiographs were checked for every patient. Only the patients with spondylolisthesis of grade I (1–24% of the vertebral body has slipped forward over the body below), segmental angulation <10°, and segmental movement less than 3 mm that was measured with flexion-extension radiographs were recruited, if any segmental instability was found. (4) Agreed to elect TF PELF or TF PELD over other spinal surgeries. (5) Failure to conservative treatment for at least 12 weeks, including but not limited to oral medication, epidural steroid injection, and physical therapy. Exclusion criteria were as follows: (1) patients who had significant spinal deformity or spinal instability and needed fusion or transferred to open surgery or lumbar interbody fusion. (2) Patients who cannot tolerate or did not agree to the surgery or did not agree to be followed up. (3) Patients with systematic infection, bleeding diathesis, or a high risk of bleeding. (4) Patients who cannot accept MRI scanning because of contraindication. (5) Patients with mental illness and who were uncooperative.

### 2.2. Surgical Technique

**Figure 3:**
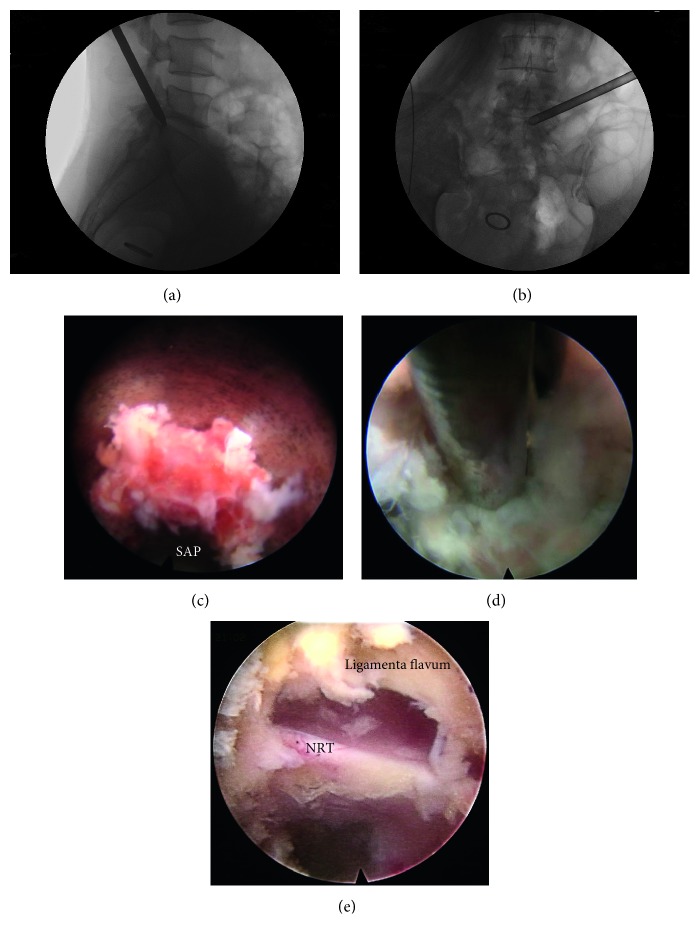
Imaging data during surgeries. (a) The tip of the guide bar lay at the posterior rim of the upper endplate of the SAP facet of the distal vertebrate in the lateral view. (b) The tip of the guide bar lay at the medial pedicle line in the anterior-posterior view. (c) Cutting SAP with a trephine under endoscope. (d) Removing the herniated lumbar disc mass under endoscope during procedure. (e) The operation area after the herniated mass was removed. SAP = superior articular process, NRT = nerve root.

All the surgeries were performed by two senior and experienced surgeons (Dr. Zhan and Dr. Xu) in TF PELD and foraminoplasty. All procedures were performed following the standard TF PELF and TF PELD technique with the transforaminal endoscopic spine system (Joimax GmbH, Karlsruhe, Germany). Patients were on the lateral position on an operating table on the contralateral side. The C-arm fluoroscopy technique was used to help surgeons determine the affected discs and pedicle and to draw a line from the midpedicular annulus to the facet lateral margin and the extension to the body surface. The skin entry point from the midline was 10–12 cm. After subcutaneous infiltration of local anesthesia with 1.0–1.5 mL 0.5% lidocaine, the subsequent steps were performed sequentially: (1) An 18-gauge needle was inserted to reach the lower segmental SAP under fluoroscopic guidance with a puncture angle of about 15° until the needle tip reached the posterior rim of the SAP of the distal vertebrate at the lateral view and the medial pedicle line at the anterior-posterior view. (2) After the stylet was retreated, another 20 mL 0.5% lidocaine was injected through the needle for adequate anesthesia. A guide wire was inserted on the same direction of the needle and a 0.8 cm in diameter incision was made, followed by a serial dilation, and a working channel was rotated into the guide wire in succession. (3) Replacing the guide wire and dilation with a guide bar (Figures 3(a) and 3(b)).

The foraminoplasty was individualized for each situation, and the surgeons decided to perform foraminoplasty depending on the operation location and experience. In cases where the working cannula could not be placed near the disc fragment due to the anatomical barrier, especially the SAP, leading to the inability of transforaminal endoscopic access to the dural sac or nerve root in the spinal canal, foraminoplasty also would be carried out to allow the working cannula access near the herniated disc [22]. Furthermore, less than 1/3 of cartilage of the SAP would be removed to maintain stability [23]. If no foraminoplasty was needed, the surgeons navigated the guide bar over the SAP of the distal vertebrate, and the working channel was accessed through the foramen. Otherwise, the foraminoplasty would be performed as follows: a tapered cannulated obturator was inserted along the guide wire, and a cannula was placed outside the foramen and lateral border of SAP; then an endoscopic trephine was used to remove the superior part of the SAP (Figure 3(c)), undercutting facet joint, ablation of osteophytes, and partially removing the foraminal ligament from outside to inside of the foramen with an endoscopic drill, bone remears, cutting forceps, and firing laser (joimax GmbH, Karlsruhe, Germany), thus, helping the surgeons access the epidural space and allowing complete decompression of foraminal or lateral recess stenosis. After the guide bar reached the position of the operation area, the working channel was rotated in the direction of the guide bar. To remove the herniated mass (Figure 3(d)), the endoscope was introduced through the cannula, and navigation was used for all cases to confirm that compression had been cleared across to the contralateral pedicle. If dural tear occurred, a small piece of gelatin sponge would be used to seal the rip. The operation area was copiously irrigated and meticulous hemostasis was achieved at the end of all surgeries (Figure 3(e)). For postoperative management, all the patients were required to wear a lumbar back brace for approximately 3-4 weeks to limit lumbar rotation.

### 2.3. Evaluation of Postoperative Outcomes and Radiography

Postoperative symptomatic improvement was evaluated by the surgeons on the operation day, and the radiography was further examined by MRI (Figures 1 and 2). Visual analogue scale (VAS) pain rating is used for estimating pain [24], and the Oswestry Disability Index (ODI) is currently considered as the gold standard for measuring life quality along with the degree of disability with low back and/or leg pain and LDH [25]. The authors adopted VAS and ODI to estimate low back and leg pain, the disability of the patients before surgery and at postoperative day 1, day 7, month 1, month 3, month 6, year 1, and year 2, respectively. In addition, the recovery of all the patients was estimated with modified Macnab criteria at postoperative 2-year follow-up.

### 2.4. Data Analysis

Quantitative data were presented as mean ± standard deviation, and qualitative data were presented as frequency (%). The normality of the data was analyzed. Mann–Whitney *U* test was utilized for nonnormal distributed data analysis between PELF and PELD groups. The Wilcoxon test was used for the nonnormal distributed data analysis within the PELF and PELD groups and the *post hoc* test for multiple comparisons. The Macnab outcomes and incidence comparisons between the 2 groups were done with *χ*^2^ test. All data were analyzed with statistical software SPSS 19.0, and *P* value <0.05 was considered significant.

## 3. Results

### 3.1. Patients' Demographic Characteristics

Eighty-six males and fifty-four females were included in this study, and the mean age was 54.5 years in the PELF group and 54.6 years in the PELD group (*P*=0.973). The pain duration in the PELF and PELD groups was 52.1 ± 89.34 and 22.9 ± 39.67 months, respectively (*P*=0.051). Operation time was 121.7 ± 46.39 minutes in the PELF group and 108.9 ± 37.70 minutes in the PELD group (*P*=0.237). The average hospital stay was 11.06 ± 9.18 days in the PELF group and 9.08 ± 3.75 days in the PELD group (*P*=0.458). The data are presented in Table 1.

Because of the loss of contact, death resulting from other diseases, or refusal to continue the follow-up, 2 patients were lost to follow-up at postoperative month 1, 2 patients at month 3, 1 patient at month 6, and 1 patient at year 2 in the PELF group. In the PELD group, 2 patients were lost to follow-up at postoperative month 1 and 1 patient at month 3 (Table 2). Thus, the average follow-up duration was 22.0 ± 6.38 months in the PELF group and 23.1 ± 4.55 months in the PELF group (Table 1, *P*=0.181).

### 3.2. Complications

Of all included patients, 2 cases had herniation at L2-3, 1 was included in the PELF and 1 in the PELD group; 6 cases had herniation at L3-4, 5 were included in the PELF and 1 in the PELD group; 76 cases had herniation at L4-5, 29 were included in the PELF and 47 cases in the PELD group; 43 cases had herniation at L5-S1, 25 were included in the PELF and 18 in the PELD group; 3 cases had herniation at both L3-4 and L4-5 levels, 1 was included in the PELF and 2 in the PELD group; 2 cases had herniation at L3-4 and L5-S1 levels, both were included in the PELD group; and 8 cases had herniation at L4-5 and L5-S1 levels, 1 was included in the PELF group and 7 in the PELD group. The distribution of the surgery sides is also presented in Table 3. Nerve root injury occurred in 1 patient herniated at L5-S1 in the PELD group (*P*=0.908) who complained postoperatively of moderate leg pain and recovered after conservative treatment for 30 days. Another 2 patients who developed dural tears received TF PELF surgery at L4-5 (*P*=0.378), but no special postoperative complaint from the patient was reported. Other 2 cases in the PELF group and 1 case in the PELD group received the second PELD within 3 months postoperatively (*P*=0.840) due to the unrelieved symptoms and imaging data indicating residuals, and no patient required conversion to an open surgery during the 2-year follow-up.

### 3.3. Comparison between Preoperative and Postoperative Clinical Outcomes within the PELF and PELD Groups

**Figure 4:**
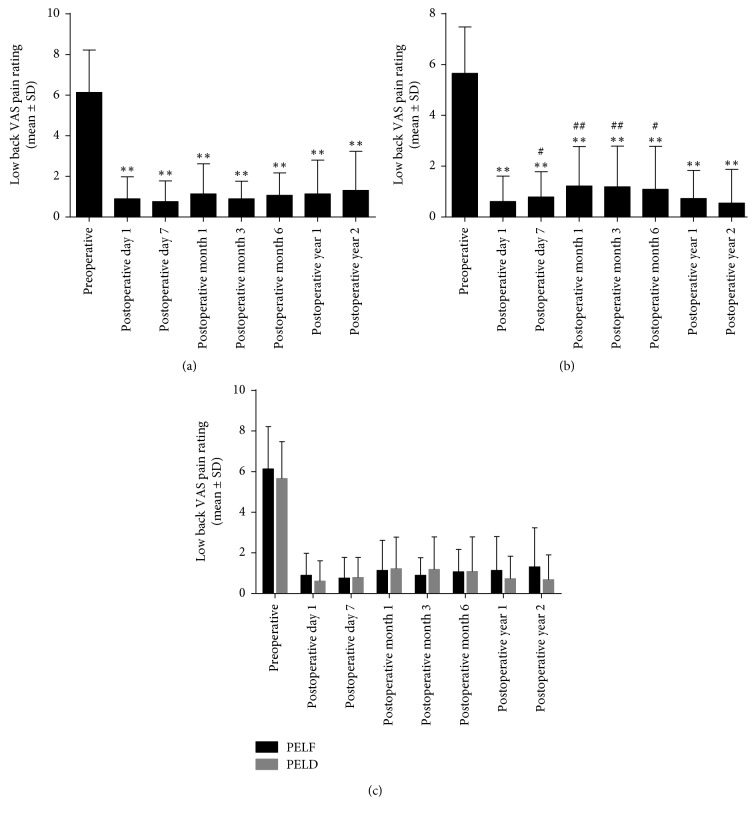
The low back pain VAS pain rating before and after TF PELF and PELD. (a) Low back pain was significantly decreased at all time points postoperatively compared with that preoperatively in the PELF group (*P* < 0.01), and no statistical difference was found between postoperative time points compared with postoperative 1-day (*P* > 0.05). (b) Low back pain decreased after TF PELD (*P* < 0.01), but increased at postoperative day 7, month 1, month 3, and month 6 compared with postoperative day 1 in the PELD group (*P* < 0.05). (c) No significant difference between the 2 groups over time (*P* > 0.05). ^*∗∗*^*P* < 0.01, compared with that preoperatively, ^##^*P* < 0.01, compared with postoperative 1-day.

**Figure 5:**
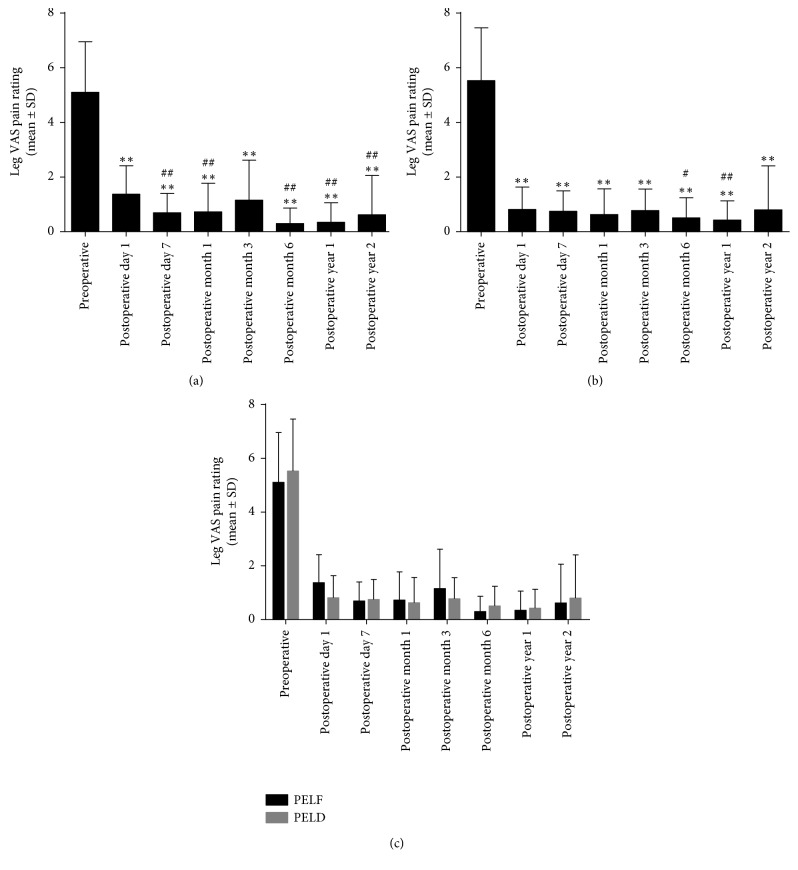
Leg VAS pain rating before and after TF PELF and PELD. (a and b) Leg VAS pain rating decreased significantly at postoperative all time points compared with that preoperatively in both PELF and PELD groups (*P* < 0.01). Compared with postoperative day 1, VAS score decreased at postoperative day 7, month 1, month 6, year 1, and year 2 in the PELF group (*P* < 0.01), and at postoperative month 6 (*P* < 0.05) and year 1 (*P* < 0.01) in the PELD group. (c) No significance between the 2 groups (*P* > 0.05). ^*∗∗*^*P* < 0.01, compared with that preoperatively; ^#^*P* < 0.05, ^##^*P* < 0.01, compared with postoperative day 1.

**Figure 6:**
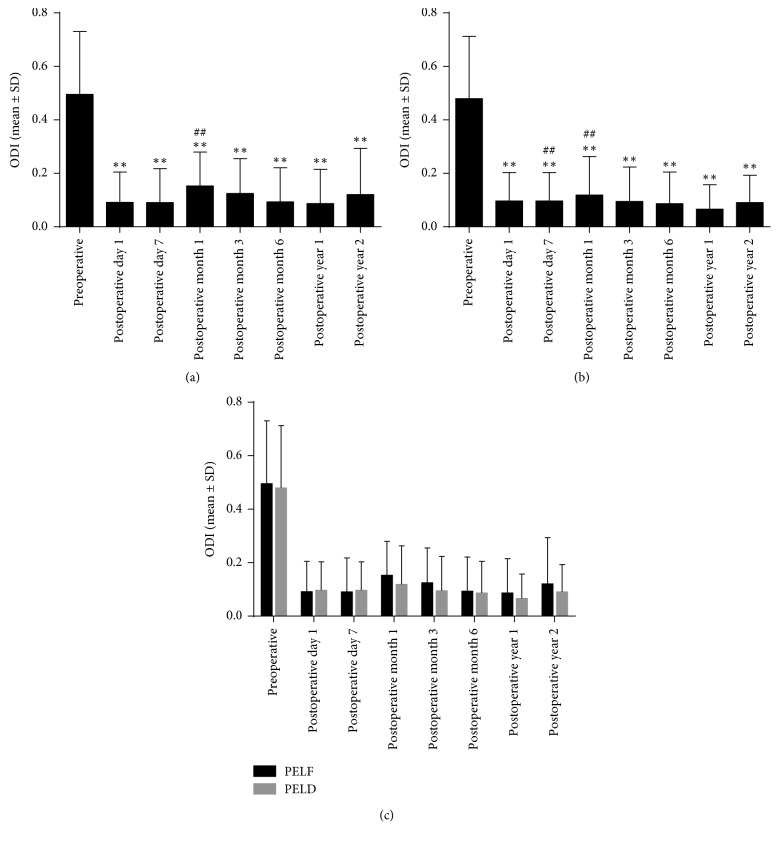
ODI before and after surgery in PELF and PELD groups. (a and b) ODI in both PELF and PELD groups significantly decreased after surgery compared with that preoperatively (*P* < 0.01). ODI at postoperative month 1 increased compared with postoperative day 1 (*P* < 0.01) in the PELF group, and increased at postoperative day 7 and month 1 in the PELD group (*P* < 0.01). (c) No significance between the 2 groups at all time points (*P* > 0.05). ^*∗∗*^*P* < 0.01, compared with that preoperatively; ^##^*P* < 0.01, compared with postoperative day 1.

VAS and ODI were utilized to estimate the surgery clinical outcomes. Compared with those preoperatively, the postoperative low back and leg VAS pain ratings and ODI scores significantly decreased over time in both groups (Figures 4–6, *P* ≤ 0.001, *P* ≤ 0.001, *P* ≤ 0.001). To further analyze the postoperative recovery of the patients, VAS and ODI scores at postoperative day 7, month 1, month 3, month 6, year 1, and year 2 were compared with postoperative day 1. We found that the postoperative change of low back VAS was not significant in the PELF group (*P*=0.948). However, low back VAS score increased at postoperative day 7 (*P*=0.046), month 1 (*P*=0.001), month 3 (*P*=0.001), and month 6 (*P*=0.014) in the PELD group. Leg VAS decreased significantly at nearly all time points postoperatively in the PELF group (*P* < 0.01) and decreased at postoperative month 6 (*P*=0.013) and year 1 (*P*=0.004) in the PELD group. ODI score increased at postoperative month 1 (*P*=0.007) in the PELF group and was increased at postoperative day 7 (*P*=0.007) and month 1 (*P*=0.001) in the PELD group.

At the final stage of the follow-up, modified Macnab criteria were used to evaluate the recovery at postoperative year 2 for the remaining 131 patients. In the PELF group, 24 cases reported “excellent” (42.9%), 21 cases reported “good” (37.5%), 6 cases reported “fair” (10.7%), and the other 5 cases reported “poor” (8.9%). In the PELD group, 38 cases reported “excellent” (50.7%), 30 cases reported “good” (40.0%), 5 cases reported “fair” (6.7%), and the remaining 2 cases reported “poor” (2.6%). Hence, the satisfactory rate reached 80.4% in the PELF group and 90.7% in the PELD group (Table 4).

### 3.4. Comparison of Clinical Outcomes between the PELF and PELD Groups

To further determine whether the injury from the TF PELF procedure would deteriorate the clinical outcomes compared with TF PELD for LDH patients, low back and leg VAS pain ratings, ODI and Macnab outcomes were compared between the 2 groups (Figures 4–6). No significant difference of low back and leg VAS pain rating was found between the PELF and PELD groups (*P*=0.654, *P*=0.722) before and after operation. Moreover, no statistical significance of ODI (*P*=0.238) and Macnab (Table 4, *P*=0.310) outcomes was found between the 2 groups.

## 4. Discussion

This is a retrospective study to explore a clinical question of whether the damage during the TF PELF procedure would deteriorate the clinical outcomes compared with the TF PELD. Sixty-two LDH-diagnosed patients who received TF PELF and 78 patients who received TF PELD were included for the 2-year follow-up. We found that low back and leg pain VAS pain ratings and ODI scores significantly decreased in both the PELF and PELD groups after surgery, although a fluctuation was observed during the follow-up period. The satisfactory rate was evaluated with modified Macnab criteria at postoperative year 2, which reached 80.4% in the PELF group and 90.7% in the PELD group. However, no significant difference between the two groups of low back and leg VAS, ODI, or satisfactory rate was recorded.

The transforaminal PELD procedure is being developed these years, and the indications of transforaminal PELD are being expanded with the invention and development of the instruments, such as ultrathin high-speed surgical drill, bone remears, cutting forceps, and firing laser. In addition to LDH, this technique can also be utilized to treat lumbar disc stenosis [26], spondylolisthesis [27], migrated recurrent disc herniation, foraminal and extraforaminal LDH [28, 29], and large disc herniations at high levels under local anesthesia [30]. The PELF is a second stage following PELD, which is used to enlarge the foramen with high-speed drill and/or trephine [16]. Both transforaminal PELD and PELF have been reported safe and effective for LDH patients, and the satisfactory rate reached over 90% in some studies [11, 12], but no study compared the effectiveness of PELD with and without foraminoplasty to determine whether foraminoplasty would severely affect clinical outcomes. The satisfactory rate of the PELD and PELF groups in the present study was 80.4% and 90.7%, which was lower than the data reported above, but was in accordance with the study reported by Nellensteijn et al. [9]. Moreover, 3 patients received a second surgery because of residuals, 2 cases in the PELF group and 1 case in the PELD group, whereas in this study, the residual rate was 2.1%, which is higher than that reported as 1.2% [8].

Recurrent herniation was defined as (1) patients with a successful PELD confirmed by a pain-free interval of at least 1 month; (2) reappearance of the initial symptoms and MRI evidence of recurrent herniation on the same level [31]. Two patients acquired dural tear in the PELF group, but no recurrence was revealed in all patients, so the incidence was lower than the previously published data [8, 9, 32, 33].

Despite the evolution, transforaminal PELD cannot be adopted in all patients due to narrow foraminal area and high iliac crest hindered by the L5 transverse process. It was reported that transforaminal PELD could be performed at the L4-5 level in 94.4% (right) and 90.4% (left) patients and only 24.1% and 19.2% at the L5-S1 level [34]. The patients who performed the interlaminar approach were not included here. However, foraminoplasty was performed in 30 cases who had herniation at the level of L4-5 and in 25 cases who had herniation at the level of L5-S1 because of high iliac crest in this study; thus, 65.1% patients who had herniation at L4-5 received TF PELD, and 46.8% patients needed foraminoplasty for larger space for endoscope navigation at L5-S1.

The disadvantages of PELF were reported as more bleeding and pain, longer operation time, prolonged postoperative recovery time, needing more expensive equipment, and higher risk of heat-damage to the surrounding spinal nerves, including neural injury [14, 21, 35]. However, we did not find any statistical difference of operation time, number of days staying at the hospital, or incidence of complications between the two groups, which might relate to both surgeons being skilled at the procedures. Therefore, we consider that foraminoplasty might extend the operation time or have a higher risk of injuring the nerve root but was not significant in this study. Although some studies reported open or closed CSF, fistulas did not readily occur in PELD because of limited access and was not recommended to attempt any dural repair after dural tear occurred [8]. The surgeon in this study sealed the dural rip with gelatin sponge intraoperatively for the patients who had dural tear to prevent postoperative hypocranial pressure symptoms. Besides, because of the irrigation during the procedure, the authors could not record the bleeding volume accurately, so the bleeding volume was not analyzed.

VAS and ODI were evaluated for all patients at each visit during the follow-up. We found that low back and leg VAS pain ratings and ODI scores decreased at postoperative day 1 compared with those preoperatively in both PELD and PELF groups, but both VAS and ODI changed significantly compared with postoperative day 1, suggesting that the symptoms of the patients would fluctuate during postoperative recovery. Low back VAS pain rating increased within 6 months postoperatively compared the first day after operation was observed in the PELD group but not in the PELF group. We postulated that it might be related with a greater range of working channel motion during the procedure in the PELD group, thus causing more damage of the peripheral spinal muscle and even local edema of nerve root, which may extend the recovery period. Nerve root injury occurred in 1 patient in the PELD group, and dural tears occurred in 2 patients in the PELF group, indicating that foraminoplasty did not result in more nerve root or ganglion, but caused complications such as dural tears, but the difference was not significant. No difference was found in Macnab outcomes between the two groups. Therefore, we considered that both TF PELD and PELF were effective and comparable for LDH treatment.

This study has some limitations. (1) Dynamic flexion-extension radiographs were not used to assess stability and hidden dynamic instability after surgeries, especially in the foraminoplasty group. (2) This is a retrospective study without controls from open discectomy, and no valid evidence from randomized controlled trials on the effectiveness of TF PELD and PELF was provided. (3) Randomized controlled trials with longer-term follow-ups compared with other surgical techniques are needed in the future. (4) Despite the LDH-diagnosed patients who reached the inclusion criteria were included in this study, the indication for accepting PELF and PELD is different, the surgeons decided to perform foraminoplasty mainly depending on operation location and experience. If the working cannula could not access the disc fragment due to the anatomical barrier, the foraminoplasty would also be performed. (5) The dimension of foramens of PELF and PELD groups were not recorded in this study, and most of the patients did not receive postoperative CT examine besides MRI, so the authors did not compare pre and postoperative foramens.

## 5. Conclusions

Both procedures are demonstrated as safe and effective for the treatment of LDH, and the clinical outcomes of TF PELF and PELD are comparable for LDH treatment. TF PELF would not deteriorate prognosis compared with PELD. However, because of the limitations of the present study, further randomized controlled trials are needed to explore the prognosis of the two procedures in future.

## Figures and Tables

**Table 1:** Comparisons of basic information between PELF and PELD groups.

Values	PELF group (*n*=62)	PELD group (*n*=78)	*P*
Female	28	26	0.153
Male	34	52	
Mean age (year)	54.5 ± 15.26	54.6 ± 13.63	0.973
Pain duration (month)	52.1 ± 89.34	22.9 ± 39.67	0.051
Operation time (minute)	121.7 ± 46.39	108.9 ± 37.70	0.094
Hospital stay (day)	11.06 ± 9.18	9.08 ± 3.75	0.458
Follow-up duration (month)	22.0 ± 6.38	23.1 ± 4.55	0.181

**Table 2:** Time points of the patients lost to follow-up.

Time	PELF group	PELD group
1 month	2	2
3 months	2	1
6 months	1	0
2 years	1	0
Total	6	3

**Table 3:** The distribution of surgery levels and sides.

Groups	PELF		PELD	
Levels	Sides			
	Left	Right	Left	Right
L2-3	1	0	1	0
L3-4	4	1	1	0
L4-5	13	16	23	24
L5-S1	17	8	10	8
L3-4 and L4-5	1	0	2	0
L3-4 and L5-S1	0	0	0	2
L4-5 and L5-S1	1	0	5	2
Total	37	25	42	36

**Table 4:** Macnab outcome evaluated at the final visit (postoperative year 2) of the follow-up.

Groups	Excellent	Good	Fair	Poor	Total	*P*
PELF	24 (42.9%)	21 (37.5%)	6 (10.7%)	5 (8.9%)	56 (100%)	0.329
PELD	38 (50.7%)	30 (40.0%)	5 (6.7%)	2 (2.6%)	75 (100%)	

Excellent: free of pain and deficit, without restriction of mobility; good: residual symptoms or deficits not impeding a normal life; fair: some improvement in functionality but remained handicapped; poor: no improvement at all.

## Data Availability

The data used to support the findings of this study are available from the corresponding author upon request.
